# Variants influencing age at diagnosis of HNF1A-MODY

**DOI:** 10.1186/s10020-022-00542-0

**Published:** 2022-09-14

**Authors:** Agnieszka H. Ludwig-Słomczyńska, Michał T. Seweryn, Piotr Radkowski, Przemysław Kapusta, Julita Machlowska, Stepanka Pruhova, Daniela Gasperikova, Christine Bellanne-Chantelot, Andrew Hattersley, Balamurugan Kandasamy, Lisa Letourneau-Freiberg, Louis Philipson, Alessandro Doria, Paweł P. Wołkow, Maciej T. Małecki, Tomasz Klupa

**Affiliations:** 1 Center For Medical Genomics OMICRON, Jagiellonian University Medical College, Kraków, Poland; 2 Department of Pharmacogenomics, The Ohio State University, Columbus, OH USA; 3 Department of Metabolic Diseases, Jagiellonian University Medical College, Kraków, Poland; 4 Department of Pediatrics, Charles University in Prague, Second Faculty of Medicine and University Hospital Motol, Prague, Czech Republic; 5 Institute of Experimental Endocrinology, Biomedical Research Center, Slovak Academy of Sciences, Bratislava, Slovakia; 6 Department of Genetics, Hôpital Pitié-Salpêtrière, Paris, France; 7 Exeter Medical School, Exeter, UK; 8 Kovler Diabetes Center, University of Chicago, Chicago, IL USA; 9 Joslin Diabetes Center, Harvard Medical School, Boston, MA USA

**Keywords:** Age at disease onset, Diabetes, GWAS, HNF1A-MODY

## Abstract

**Background:**

HNF1A-MODY is a monogenic form of diabetes caused by variants in the *HNF1A* gene. Different *HNF1A* variants are associated with differences in age of disease onset, but other factors are postulated to influence this trait. Here, we searched for genetic variants influencing age of HNF1A-MODY onset.

**Methods:**

Blood samples from 843 HNF1A-MODY patients from Czech Republic, France, Poland, Slovakia, the UK and the US were collected. A validation set consisted of 121 patients from the US. We conducted a genome-wide association study in 843 HNF1A-MODY patients. Samples were genotyped using Illumina Human Core arrays. The core analysis was performed using the GENESIS package in R statistical software. Kinship coefficients were estimated with the KING and PC-Relate algorithms. In the linear mixed model, we accounted for year of birth, sex, and location of the HNF1A causative variant.

**Results:**

A suggestive association with age of disease onset was observed for rs2305198 (p = 2.09E−07) and rs7079157 (p = 3.96E−06) in the *HK1* gene, rs2637248 in the LRMDA gene (p = 2.44E−05), and intergenic variant rs2825115 (p = 2.04E−05). Variant rs2637248 reached nominal significance (p = 0.019), while rs7079157 (p = 0.058) and rs2825115 (p = 0.068) showed suggestive association with age at diabetes onset in the validation set.

**Conclusions:**

rs2637248 in the *LRMDA* gene is associated with age at diabetes onset in HNF1A-MODY patients.

**Supplementary Information:**

The online version contains supplementary material available at 10.1186/s10020-022-00542-0.

## Background

Maturity-onset diabetes of the young (MODY) is a monogenic form of diabetes that accounts for approximately 1–2% of diabetes cases. One of the most common subtypes is hepatocyte nuclear factor-1-alpha (HNF1A)-MODY, caused by variants of the *HNF1A* gene. HNF1A-MODY is characterized by a severe deficit in insulin secretion and a decreased renal threshold for glucose reabsorption. Because HNF1A-MODY is associated with sustained sulfonylurea sensitivity, correct recognition of the disease subtype at the time of diagnosis is important for optimal treatment selection (Kavvoura and Owen 2014; Timsit et al. 2016).

Located on chromosome 12, the *HNF1A* gene encodes a 631-amino-acid transcription factor composed of an N-terminal dimerization domain, a DNA-binding domain and a C- terminal transactivation domain. HNF1A functions either as a homodimer or as a heterodimer in association with hepatocyte nuclear factor-1-beta. There are three different isoforms of HNF1A, which vary in their tissue-specific expression and transcriptional properties. One of the main sites of HNF1A expression is in the pancreatic islet beta-cells, where it affects regulation of other islet-specific transcription factors (Boj et al. 2001). *HNF1A* is also expressed in the liver, intestine and kidney. More than 400 causative variants segregating with MODY have been identified across the *HNF1A* gene, having diverse effects on protein properties and cellular phenotypes (Thomas et al. 2002; Yamagata 2014; Balamurugan et al. 2016; Colclough et al. 2013). The vast majority of them are missense variants occurring mostly in the dimerization or DNA-binding domains, or truncating variants occurring mostly in the transactivation domain (Ellard and Colclough 2006).

HNF1A-MODY is typically diagnosed in the second or third decade of life. However, there is wide variability in age at onset, which appears to be influenced by both the location and type of *HNF1A* variant (Bellanné-Chantelot et al. 2008; Tatsi et al. 2013). For example, truncating or missense variants in the dimerization or DNA—binding domain and variants that affect all three *HNF1A* isoforms have been associated with earlier age at diagnosis (Bellanné-Chantelot et al. 2008; Locke et al. 2018). However, while the type and location of variants are major predictors of the clinical course of the disease, substantial differences are observed even among related individuals in the same generation carrying the same variant (Hattersley 1998; Porter and Barrett 2005). Other variables proven to affect age at onset include environmental factors and variation in genes other than *HNF1A*. We have previously shown that age at onset of diabetes in families with *HNF1A* causative variant is influenced by both familial factors (including modifying genes) and the parent of origin. Among individuals with maternal inheritance of the causative variant, diabetes had presented by age 15 years in 57 ± 8% of those with in utero exposure to diabetes compared with none of those without in utero exposure (Klupa et al. 2002).

Nonetheless, the parent-of-origin effect only partially explains the genetic (familial) influence on the variability in age at diagnosis of HNF1A-MODY. Identification of other genetic factors influencing the age of HNF1A-MODY presentation could provide numerous clinical benefits. For example, it could inform the timing of initiation or intensification of diabetes monitoring in *HNF1A* variant carriers, thereby saving time and resources and reducing the burden on affected families. It may also foster further research aiming to delay the development of the disease in variant carriers. Finally, the relevance of any findings may extend beyond HNF1A-MODY to more common forms of diabetes (Klupa et al. 2012).

In the present study, we aimed to identify for the first time the genetic variants influencing age at onset of the phenotypic presentation of HNF1A-MODY by GWAS method. We also examined whether common variants in HNF1A target genes affected age at onset of the disease.

## Materials and methods

### Study participants

We conducted a genome-wide association study (GWAS) in confirmed carriers of *HNF1A* variants associated with diabetes. Patients were enrolled in six countries: Czech Republic, France, Poland, Slovakia, the UK and the US (Boston). A group of 121 participants from the US (Chicago) served as a validation set. Given the results of previous GWAS studies on MODY patients (Locke et al. 2018) we set out to perform the GWA study on a combined cohort of MODY patients. Our basic assumption was to reach the level of at least 500 individuals (which is larger than any previous study). At the same time, the power estimates were heavily dependent on the size and structure of families, therefore we do not present these estimates in the manuscript. Detailed information about number of families included in the analysis as well as family structures is provided in Additional file 1: Data S1. Among the study participants were 309 probands and 534 of their family members (coming from 175 families). Patients recruitment was based on local MODY registries of participating centers. For the leading center the MODY registry was established in 2004 and maintained since that time. Although the selection criteria during recruitment differed among participating centers, retrospectively all centers agreed upon following criteria as a “minimal ones” for the patients to have been screened towards MODY:

- age of diagnosis below 40 years
- a family history of diabetes in two or more generations
- lack the characteristics of type 2 diabetes (marked obesity, acanthosis nigricans).
- lack the characteristics of type 1 diabetes (no islet autoantibodies – if measured, low or no insulin requirements more than 5 years after diagnosis)

Patients meeting MODY criteria in whom causative mutation in *HNF1A* was identified were included into the study. The causative mutation was indicated for each patient by local clinicians. Most of the diagnoses were identified based on *HNF1A* sequencing and pedigree analysis. The diabetes was diagnosed according to World Health Organization Criteria. The study was approved by local bioethics committees. All participants provided written informed consent.

### Genotyping and quality control

DNA was extracted in the country of sample origin. Samples were gathered at the Center for Medical Genomics OMICRON in Kraków, Poland where they were genotyped using Illumina Human Core arrays (Illumina, San Diego, CA, USA) according to the manufacturer’s instructions. Quality control (QC) was performed as described previously (Ludwig-Słomczyńska et al. 2020) and is presented in detail in the Additional file 1: Data S2. A total of 282,876 autosomal polymorphisms were eligible for the analysis. The genotyping of the replication set was performed by the Molecular Phenotyping and Genotyping Core of the Joslin Diabetes Center, USA.

### Imputation

The QC-filtered genotype data were checked against the reference panel of the Haplotype Reference Consortium (HRC; version r1.1 2016) (McCarthy et al. 2016) using the HRC/1000G Imputation Preparation and Checking Tool (version 4.2.9) (Farmaki et al. 2017) to exclude strand-coding issues during the imputation step. Imputation of QC-filtered genotypes was performed using Michigan Imputation Server (with Minimac3) (Das et al. 2016) and the HRC reference panel. Phasing was performed with SHAPEIT (version 2.r790) (O’Connell et al. 2014). The Minimac3 output dosage files were converted to hard calls in PLINK.

### Genotype and tissue expression project

Tissue-specific RNA sequencing and phenotype data were acquired from the Genotype and Tissue Expression (GTEx) project via the database of Genotypes and Phenotypes (dbGaP) Project #5358 (dbGaP accession number: phs000424). For further details see Lonsdale et al. (Lonsdale et al. 2013) and the GTEx website (http://www.gtexportal.org/home/documentationPage). Testing for expression quantitative trait loci (eQTLs) was performed via the GTEx online search tool (Aguet et al. 2017).

### Statistical analysis

The core analysis was performed using the GENESIS package in R statistical software. Kinship coefficients were estimated with the KING and PC-Relate algorithms. The effect size was estimated via the linear mixed model approach with correlation structure described by the kinship matrix. The age of onset was log-transformed to better fit the Gaussian distribution. In the linear mixed model, we also accounted for date of birth, sex and location of the *HNF1A* causative variant. Robustness of the results was evaluated by adding the first five PC’s to the ‘basic model’. In the main analysis, SNPs were coded as allele dosages. In the model with interaction between a SNP and the location of causative variant, SNPs were coded as allele dosages and the location of the causative variant was treated as a factor. The list of HNF1A target genes was based on the Transcriptional Regulatory Relationships Unraveled by Sentence-based Text Mining (TRRUST) database (Han et al. 2015).

The analysis on the validation set was performed with the use of linear mixed models as implemented in the package ‘coxme’ in R. The trait (age of onset) was log-transformed to avoid the inflation of estimated due to overdispersion. In the model, we included: current age, sex and location of the causative *HNF1A* variant. The kinship matrix was included as provided from the pedigree data by the University of Chicago. The p-values refer to the ANOVA-type test with the likelihood ratio test statistics for model comparison.

### Data and resource availability

The datasets generated and/or analyzed during the current study are available from the corresponding author upon reasonable request.

## Results

### Clinical characteristics

**Fig. 1:**
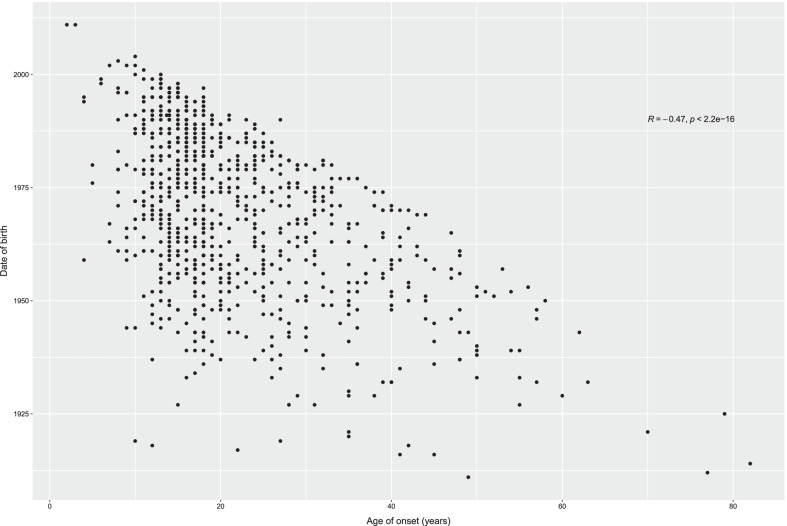
Dependence of age at disease diagnosis on date of birth

**Fig. 2:**
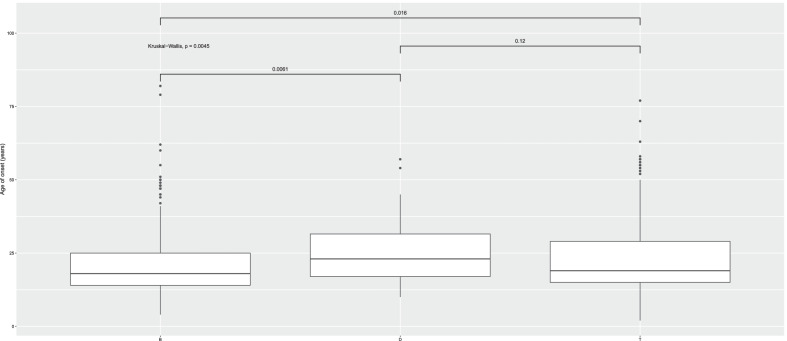
Location of HNF1A variant and age at diagnosis in patients with variants in the DNA-binding (B), dimerization (D) or transactivation (T) domains of HNF1A

Study participants consisted of 843 carriers of *HNF1A* causative variants, including 309 probands and 534 of their family members (coming from 175 families). Salient clinical characteristics of study subjects are presented in Table 1 and Additional file 1: Data S1. The variants observed among studied participants along with their ACMG classification are listed in Additional file 4: Table S1. 216 variants were observed among studied patients. 102 of them were pathogenic according to ACMG criteria, 37 were likely pathogenic, 72 of uncertain significance (VUS) and only 5 were benign. Among VUS only 4 had “BS” mark indicating evidence of benign impact, while 5 “BP” mark, which would support their benign characteristics. Given progress in MODY research, improvements in clinical diagnostic criteria and new diagnostic methods, we reasoned that date of birth should be included in the statistical model analyzing the determinants of age at onset. As expected, there was a strong negative association between age at onset and date of birth (Fig. 1), i.e., the younger was the proband, the higher were his/her chances of earlier diagnosis. Since previous results suggested that location of the causative variant in the *HNF1A* gene might influence age at onset, we grouped observed variants according to their location in the dimerization, DNA-binding or transactivation domain (Awa et al. 2011). We then tested for association between age of onset and location of the causative variant (Fig. 2). We found out that location of the variant in the dimerization domain (D) domain would advance age of disease onset the most, while mutation in the DNA-binding domain (B) would lead to earliest disease presentation (in comparison to transactivation (T) or dimerization (D) domains).

**Table 1:** 843 HNF1A variant carriers characteristics in the discovery cohort

Country	N	Males, %	Mean age (SD), years	Mean age at onset (SD), years	No. of causative variants per HNF1A domain		
					DNA-binding	Dimeri-zation	Trans-activation
Czech Republic	89	34	43.63 (15.53)	20.04 (9.28)	50	2	37
France	277	32	44.34 (15.81)	21.79 (10.32)	124	20	133
Poland	85	25	47.09 (15.29)	24.30 (10.54)	51	6	28
Slovakia	59	49	42.95 (15.06)	19.96 (9.39)	41	2	16
UK	242	30	56.14 (18.24)	22.00 (11.55)	90	6	146
USA	91	50	64.89 (18.13)	25.11 (15.36)	51	6	34
p-value	–	7.12E−04	< 2E−16	0.0127	1.80E−06		

*HNF1A* hepatocyte nuclear factor-1-alpha, *SD* standard deviation

### GWAS of interaction with the causative variant

Common genetic variants influencing the age at onset of HNF1A-MODY were sought through a genome-wide study. Since the effects of the modifying genetic variants may depend on the location of the causative *HNF1A* variant, the GWAS model included the interaction of *HNF1A* domain (bearing causative variant) x SNP interaction. The top 10 variants (analyzed on directly genotyped variants), based on the joint 2 d.f. p value, are shown in Table 2. The strongest association was observed for two SNPs (rs2305198 and rs7079157) at the HK1 (hexokinase) locus, with p values of 2.09 × 10^–7^ and 3.96 × 10^–6^. Another SNP (rs9939578 at the Trans-Golgi Network Vesicle Protein 23 Homolog A [TVP23A]) had p-value 4.66 × 10^–6^, while the remaining 7 SNPs had p-values < 1 × 10^–5^. For five of the SNPs (rs282115, rs2637248, rs6848074, and rs4949632, rs11081446), there was a major contribution of an *HNF1A* variant x SNP interaction to the overall significance. In most instances, the SNP had a stronger effect on the age of onset if the causative variant was localized in the dimerization rather than in the transactivation domain. The standard GWAS with no interaction analysis was also performed. The results are presented in Additional file 1: Data S3.

**Table 2:** Results of GWAS analysis depending on causative variant location in the discovery cohort

SNP ID	Gene	MAF	Effective allele	Est. G	Est G:D domain	Est G:T domain	GxE p-value	Joint p-value
Ela powina rs2305198	*HK1*	0.38	C	− 0.068	− 0.205	− 0.050	4.50E−02	2.09E−07
rs7079157	*HK1*	0.23	C	− 0.067	− 0.245	− 0.055	5.25E−02	3.96E−06
rs9939578	*TVP23A*	0.12	G	− 0.049	0.213	− 0.151	1.32E−03	4.66E−06
rs10865231	*STON1-GTF2A1L*	0.26	G	− 0.062	− 0.115	− 0.068	2.04E−01	1.82E−05
rs2825115	Intergenic	0.44	C	− 0.064	− 0.295	0.117	1.14E−05	2.04E−05
rs2637248	*LRMDA-215*	0.14	G	*− 0.141*	− 0.218	0.208	3.97E−05	2.44E−05
rs6848074	*LOC101929199*	0.25	G	− 0.003	0.427	− 0.067	6.04E−06	2.27E−05
rs4949632	*ST6GALNAC3*	0.34	G	0.056	− 0.409	− 0.113	1.03E−05	2.84E−05
rs210307	*AL163953.3*	0.23	C	0.142	0.028	− 0.098	7.16E−02	2.86E−05
rs11081446	Regulatory region variant	0.48	G	0.116	− 0.158	− 0.167	3.20E−05	3.07E−05

*D* dimerization domain, *GWAS* genome-wide association study, *ID* identifier, *MAF* minor allele frequency, *SNP* single-nucleotide polymorphism, *T* transactivation domain. Est G. main effect of the risk allele estimated in the Linear Mixed Model; Est G:D the estimated interaction effect of the risk allele and the indicator variable of the dimerization domain; Est G:T the estimated interaction effect of the risk allele and the indicator variable of the transactivation domain; GxE p-value: the p-value for the null hypothesis that the Est G:T and Est G:D equal zero; Joint p-value the p-value for the null hypothesis that Est G, Est G:T and Est G:D equal zero

### eQTL analysis for identified variants

**Fig. 3:**
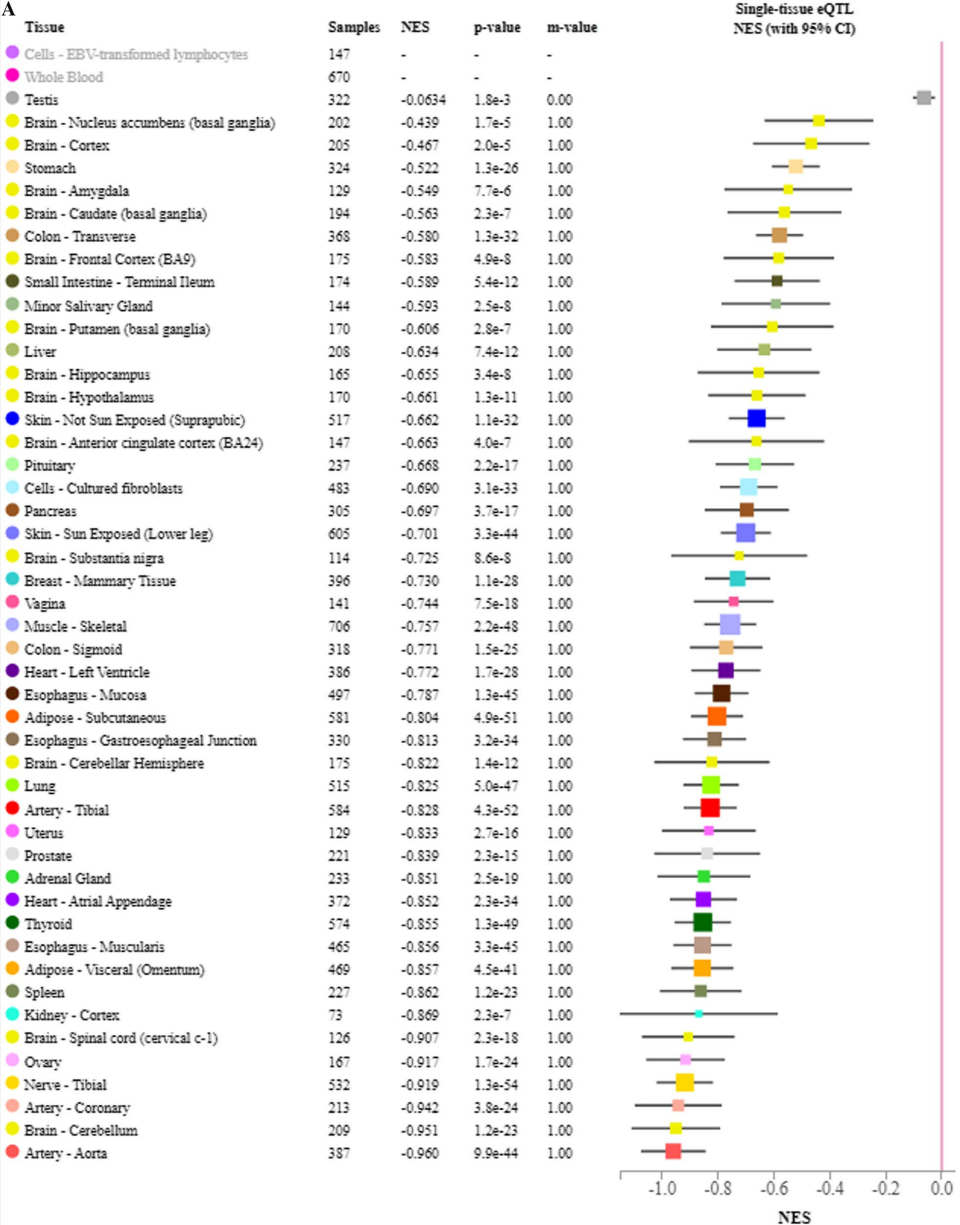

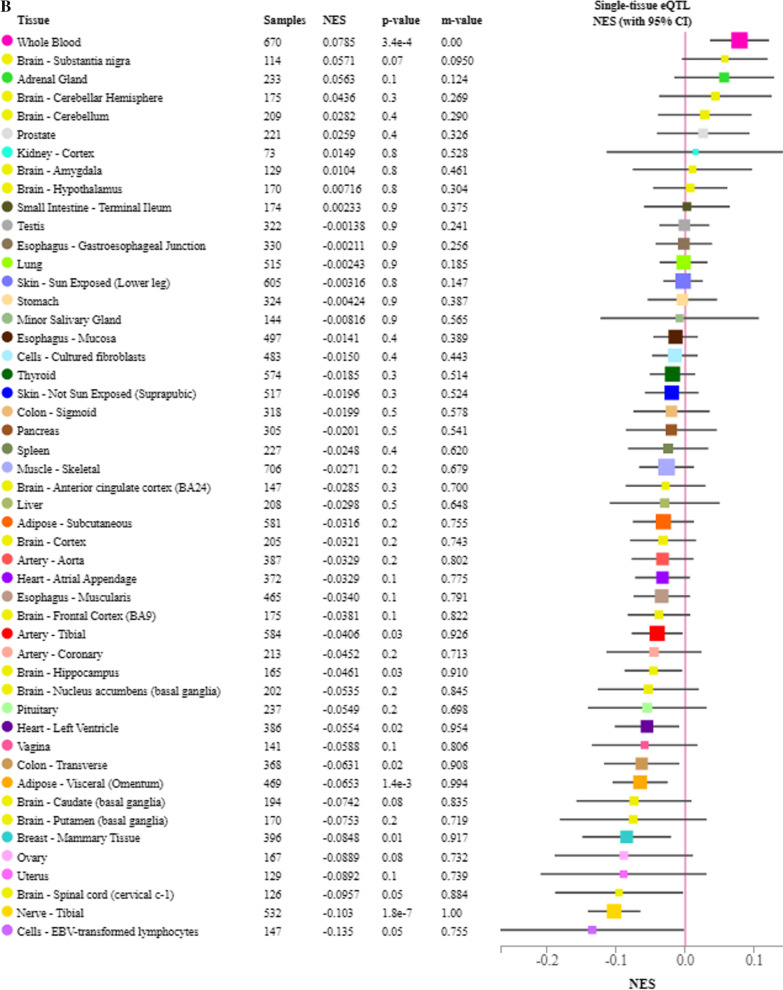

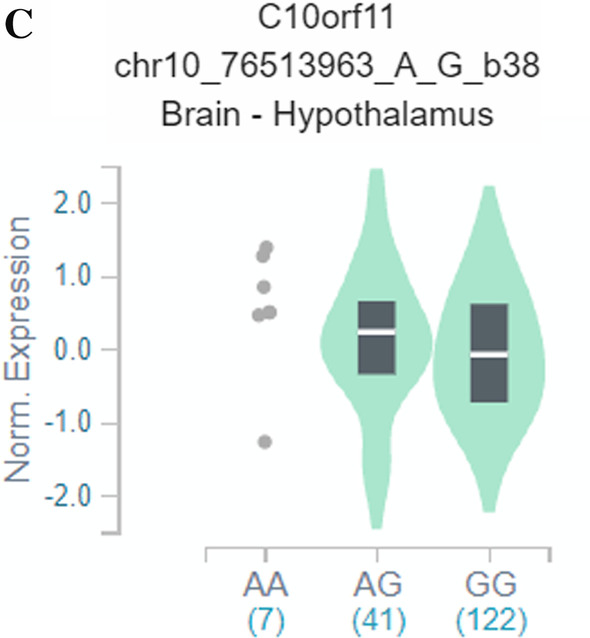
eQTL analysis for HK1 SNP rs2305198 **A** and STON1-GTF2A1L SNP rs10865231 **B** and LRMDA SNP rs2637248 (**C**); *CI* confidence interval, *eQTL* expression quantitative trait locus, *NES* normalized effect size, *SNP* singlE−nucleotide polymorphism

We next used GTEx data to examine whether the identified variants influenced the expression of the genes in which they are located. The two *HK1* SNPs, rs2305198 and rs7079157, are in linkage disequilibrium (LD) and the former is more frequent in the European population. In the GTEx database, the minor allele of rs2305198 was associated with higher *HK1* expression in whole blood and lower *HK1* expression in many other tissues, including those of the cardiovascular system (heart and aorta) and the nervous system (some brain regions and tibial nerve) (Fig. 3A). The minor allele of rs10865231 was significantly associated with lower expression of *STON1-GTF2A1L* across many human tissues (Fig. 3B). Also, the minor allele of rs2637248 was associated with higher expression of LRMDA (aka C10orf11) in several regions of the brains, including the hypothalamus (Fig. 3C). No data were available in GTEx for the other top variants.

### Validation set

Validation of the top ten signals from the GWAS was sought in a validation set consisting of 121 HNF1A-MODY participants from the University of Chicago Monogenic Diabetes Registry (www.monogenicdiabetes.org). Salient characteristics of these subjects are presented in Table 3. Due to technical difficulties, we were unable to genotype rs11081446, while rs4949634 was used as a proxy for rs4949632. Variant rs2637248 reached nominal significance for association with the age of diabetes onset (p = 0.019), while two other variants rs7079157 (p = 0.058) and rs2825115 (p = 0.068) showed suggestive association with this trait (Table 4). In all cases, similarly to the results obtained in the genome-wide study, the presence of the variant was associated with a greater delay in the age of disease presentation if the causative variant occurred in the dimerization domain as opposed to the transactivation domain.

**Table 3:** 121 HNF1A variant carriers characteristics in the validation set

Number of participants (N)	121
Males, %	27.5
Mean age (SD), years	40.8 (18.34)
Mean age at diagnosis (SD), years	18.86 (10.37)
# of causative variants in domain B	80
# of causative variants in domain D	5
# of causative variants in domain T	36

^#^number; B, DNA binding domain; D, dimerization domain; T, transactivation domain

**Table 4:** Results of GWAS analysis depending on causative variant location in the validation set

SNP ID	Gene	MAF	Effective allele	Est. G	Est G:D domain	Est G:T domain	ANOVA p-value
rs2305198	*HK1*	0.354	C	1.381	7.655	0.994	0.396
rs7079157	*HK1*	0.165	C	− 0.205	15.148	5.848	0.058
rs9939578	*TVP23A*	0.144	G	− 0.639	NA	NA	NA
rs10865231	*STON1-GTF2A1L*	0.675	G	− 0.141	9.985	3.494	0.153
rs2825115	Intergenic	0.483	C	− 0.266	15.182	3.409	0.068
rs2637248	*LRMDA-215*	0.176	A	− 1.021	− 10.563	3.684	0.019
rs6848074	*LOC101929199*	0.250	A	0.205	NA	NA	NA
rs4949634	*ST6GALNAC3*	0.359	A	1.175	NA	− 1.906	0.639
rs210307	*AL163953.3*	0.231	T	0.709	− 9.281	0.549	0.127

*D* dimerization domain, *GWAS* genome-wide association study, *ID* identifier, *MAF* minor allele frequency, *SNP* single-nucleotide polymorphism, *T* transactivation domain, Est G. main effect of the risk allele estimated in the Linear Mixed Model; Est G:D the estimated interaction effect of the risk allele and the indicator variable of the dimerization domain; Est G:T the estimated interaction effect of the risk allele and the indicator variable of the transactivation domain; GxE p-value: the p-value for the null hypothesis that the Est G:T and Est G:D equal zero; Joint p-value the p-value for the null hypothesis that Est G, Est G:T and Est G:D equal zero

### Common variants in HNF1A target genes and their influence on age at onset

Since HNF1A is a transcription factor, variation in its target genes might affect its binding affinity, and thereby influence target gene expression and ultimately the age at diabetes onset. We therefore searched the TRRUST database to identify HNF1A target genes (Han et al. 2015). For each of the target genes, variants in the intergenic regions (covering 500 kb up- and downstream) (based on imputed genotypes) were selected for the analysis. As previously, *HNF1A* causative variants were grouped according to their location in the dimerization, DNA-binding or transactivation domains. Association between common variants in target genes and age at onset that depended on causative variant location was found for 16 variants at the *AFP/AFM*, *CYP1A2*, *SLCO1B3*, *MTTP*, *CRP* and *CSRP1* loci (Table 5).

**Table 5:** Influence of common variants in HNF1A target genes on age at diagnosis

Gene	SNP ID	Effective allele	Est. G	Est. G:D domain	Est. G:T domain	GxE p-value
*AFP*/*AFM*	rs12510870	C	− 0.0658	0.0782	0.1418	0.0096
	rs16849364	G	− 0.0472	0.0322	0.1290	0.0218
	rs4640638	G	− 0.0437	0.0449	0.1211	0.0355
*CYP1A2*	rs12441817	C	0.0228	− 0.4899	− 0.0044	0.0109
*SLCO1B3*	rs10841644	C	− 0.0049	0.2745	0.0089	0.0089
	rs7965380	C	− 0.0019	0.2716	0.0073	0.0097
	rs957164	G	0.0031	0.2797	− 0.0138	0.0103
	rs2033515	G	− 0.0406	0.3366	0.0458	0.0193
*MTTP*	rs12647527	G	− 0.0063	− 0.1420	0.0376	0.0050
	rs881980	G	− 0.0025	− 0.4047	0.0365	0.0094
*CRP*	rs12094103	G	0.01945	− 0.2369	− 0.0718	0.0115
	rs3122012	C	0.0116	− 0.2509	− 0.0597	0.0123
*CSRP1*	rs7525711	G	− 0.0186	− 0.2557	0.0517	0.0063
	rs11577209	G	− 0.0115	− 0.1766	0.0833	0.0111
	rs10920205	G	− 0.0061	− 0.2557	0.0100	0.0139
	rs2038972	C	− 0.0466	0.0086	0.1344	0.0286

*D* dimerization domain, *HNF1A* hepatocyte nuclear factor-1-alpha, *ID* identifier, *SNP* single-nucleotide polymorphism, *T* transactivation domain, Est G. main effect of the risk allele estimated in the Linear Mixed Model; Est G:D the estimated interaction effect of the risk allele and the indicator variable of the dimerization domain; Est G:T the estimated interaction effect of the risk allele and the indicator variable of the transactivation domain; GxE p-value: the p-value for the null hypothesis that the Est G:T and Est G:D equal zero; Joint p-value the p-value for the null hypothesis that Est G, Est G:T and Est G:D equal zero

Main effects of the identified variants in *AFP/AFM* accelerated disease onset but the interaction effect showed delay of disease onset both if present alongside variants in the *HNF1A* dimerization domain (Est. G:D domain) or transactivation domain (Est. G:T domain). Conversely, main effect of the variant in *CYP1A2* (rs12441817) is associated with later disease onset, while its interaction with the variant present in dimerization or transactivation domain leads to earlier disease presentation. Similar effects are observed for the *CRP* variants rs12094103 and rs3122012. For *SLCO1B3*, *MTTP* and *CSRP1*, interaction effects differ between the identified variants. For example, the main effects of *CSRP1* variants rs7525711, rs11577209, rs10920205 and rs2038972 advance age of diagnosis. The first three of these *CSRP1* SNPs further advances disease onset if co-occurring with dimerization domain causative variant, whereas a compensatory effect is observed if these variants co-occur with transactivation domain causative variant. Such differences are probably explained by LD. Variants within the *AFP/AFM*, *MTTP* and *CRP* genes are in LD with one another. *CSRP1* rs7525711 and rs10920205 are in LD, while, for *CSRP1* rs11577209 and rs2038972, *D*′ is 1 and *R*^2^ is 0.5. In *SLCO1B3*, only the variants rs10841644 and rs7965380 are in LD (*D*′ = 1; *R*^2^ = 1), whereas rs957164 and rs2033515 appear to be on different haplotypes.

## Discussion

The age of onset of HNF1A-MODY is quite variable. Previous studies have shown that the location of the causative variant in the *HNF1A* gene and the parent from which the variant is inherited account for part of this variability. In this genome-wide association study, we identified several variants with suggestive evidence for a modulatory effect on age of diabetes onset in a group of 843 HNF1A-MODY patients. The modulating effect of these variants appeared to depend on the location of the *HNF1A* causative variant. We also found other variants with a potential effect on the age at onset through a candidate gene analysis focused on genes coding for transcription targets of HNF1A.

One of the variants identified (rs2637248) showed a nominally significant association with age at onset in validation group of MODY patients, supporting the veracity of this genetic effect. It is placed in an intron of the LRMDA gene (also known as C10orf11), which codes for a leucine-rich repeat protein thought to play a role in melanocyte differentiation. Variants in this gene have been associated with autosomal recessive oculocutaneous albinism 7 (OCA7), but have never been implicated in metabolic disorders (Grønskov et al. 2013). Interestingly, however, the variant associated with age at diabetes onset is also associated with differences in LRMDA expression in the brain, including the hypothalamus, where peptides controlling pigmentation, such as melanocortins and their receptors, are involved in the regulation of body weight and metabolism (Gantz and Fong 2003). Moreover, other variants of LRMDA gene were associated with metabolic phenotypes i.e. with diabetes (Spracklen et al. 2020; Vujkovic et al. 2020), triglyceride levels (Richardson et al. 2020) as well as BMI and waist-to-hip ratio adjusted for BMI in the UK Biobank analysis (Zhu et al. 2020). Moreover, another LRMDA variant (rs10824307) was shown to associate with T2D subtype, named mild obesity-related diabetes (MOD) in genomic risk score analysis (Mansour Aly et al. 2021).

Two other variants – rs2825115 and rs7079157 – showed suggestive trends of association with the outcome in the validation set that went in the same direction as the effects observed for these variants in the discovery set. SNP rs2825115, located in an intergenic region, next to a microRNA (MIR548X), shows an association with age the phenotype regardless of the type of analysis. This suggests that it might be associated with the outcome, but the effect of epistasis with the causative variant possibly augments the relationship. The other variant (rs7079157) is located in the *HK1* gene coding for the enzyme hexokinase 1. The analysis of the discovery set suggested an association with another *HK1* variant (rs2305198); this, however, was not confirmed in the validation sample. An effect of the *HK1* gene on age of HNF1A-MODY onset would not be surprising, given that HK1 catalyzes the conversion of glucose to glucose-6-phosphate in the first step of the glycolytic pathway. Associations between *HK1* variants and glycated hemoglobin (HbA1c) levels have been reported. SNPs rs7072268 and rs17476364 were found to be correlated with HbA1c levels in healthy individuals (Paré et al. 2008; An et al. 2014); however, it was suggested that the correlation between *HK1* variants and HbA1c levels might be independent of glycemic factors (Bonnefond et al. 2009; Soranzo et al. 2010; Rijksen et al. 1983; Bianchi and Magnani 1995). To date, only one study correlating *HK1* variants with HbA1c levels has included patients with diabetes (Gjesing et al. 2011). This confirmed an association of rs7072268 with HbA1c and reported a correlation with fasting cholesterol and nominal association with type 2 diabetes. rs7079157 associated with age at onset in our study is placed in *HK1* intron. It does not seem to impact regulatory elements, but GTEx data show that it is associated with HK1 expression in several tissues.

Through a candidate gene sub-analysis of the GWAS data focused on HNF1A target genes, we also showed that age at onset may be influenced by variants in several HNF1A target genes. Especially interesting in this list is the presence of the AFM gene coding for afamin – a member of the albumin family. While the physiological functions of afamin are not well understood, its overexpression in transgenic mice lead to increased body weight as well as hyperglycemia and hyperlipidemia (Kronenberg et al. 2014). A recent study of more than 20,000 individuals has suggested afamin as a potential biomarker for the identification of individuals at high risk of type 2 diabetes (Kollerits et al. 2017). As far CRP is concerned, we co-authored a multicenter European study demonstrating that the CRP serum levels were significantly lower in HNF1A-MODY than in type 1 or type 2 diabetes (Thanabalasingham et al. 2011). In that study, CRP enabled differential diagnosis of HNF1A-MODY, with a receiver operating characteristic curve-derived C-statistic for discrimination between HNF1A-MODY and other diabetes types of 0.86 (95% confidence interval: 0.83, 0.88). Subsequent studies have questioned the additional value of using high-sensitivity CRP assays in the differential diagnosis of HNF1A-MODY and familial young-onset type 2 diabetes (Bellanné-Chantelot et al. 2016). The results of our genetic study suggest that it might be worth looking into more deeply.

Our study is the first GWAS of genetic determinants of age of disease onset in HNF1A-MODY patients in a large patient group. However, this study has also some limitations. Since the study population consisted of probands, but also of their relatives, the reported age of diabetes onset for the other family member depended on the age at diagnosis of the proband. Moreover, the study population included patients from six different countries, which differed in the progress of monogenic diabetes diagnosis. Lastly, even though this study is the largest GWAS on age of disease onset to date, the sample size is still small for a GWAS analysis. This is probably the reason why most of the identified associations are suggestive (i.e., below the genome-wide threshold for significance). Still, however, we were able to confirm some of the associations from the exploratory set in a validation sample, which suggests that some of these loci are true positives deserving further research.

## Conclusions

In summary, we have identified several genetic variants that may influence the age of diabetes onset in patients with HNF1A-MODY. Identification of such association with rs2637248 located in *LRMDA* gene opens a new field of research for this gene. Furthermore, the identification of genes modifying the age of disease presentation in *HNF1A* variant carriers provides new insights into the pathophysiology of HNF1A-MODY, which may more broadly impact our knowledge of the regulation of glucose homeostasis. Finally, it may be possible to extend the findings of our study to more common forms of diabetes, especially with regard to the role of gene–gene interactions.

## Supplementary Information

**Additional file 1.** Additional data S1. 

**Additional file 2.** Additional data S2.  

**Additional file 3.** Additional data S3.  

**Additional file 4.** Additional table. 

## Data Availability

The data sets generated and analyzed during the current study are available from the corresponding author upon request.

## Abbreviations

MODY: Maturity-onset diabetes of the young
GWAS: Genome-wide association study
HNF1A: Hepatocyte nuclear factor-1-alpha
HNF1A-MODY: Maturity-onset diabetes of the young caused by a mutation in HNF1A gene
MAF: Minor allele frequency
SNP: Single-nucleotide polymorphism
