# Supplementary material for: Variants influencing age at diagnosis of HNF1A-MODY
Source: Mol Med. 2022 Sep 14;28:113. doi: 10.1186/s10020-022-00542-0 (PMC9476297; doi:10.1186/s10020-022-00542-0)
Supplement: Supplementary file 2 — Additional file 2. Additional data S2. [file 10020_2022_542_MOESM2_ESM.docx]

**Supplementary Data 2**

**SNPs Quality Control Analysis**

Genotypes were called by GenomeStudio software Genotyping module (version 2.0, Illumina Inc.). A two-step quality check (QC) of genotyped SNPs was applied. Supplementary Information Table S2_1 summarizes the SNPs quality control (QC) processing steps. First, we applied hard cut off metrics in GenomeStudio according to manufacturers’ instructions (Technical Note: Genotyping, Infinium ® Genotyping Data Analysis, [www.illumina.com](http://www.illumina.com/)) to remove SNPs with poor clustering quality metrics. Then, we removed SNPs with two or more discordant genotypes in the duplicated samples and filtered autosomal SNPs for Hardy-Weinberg disequilibrium. The remaining SNPs were exported as custom report using plink (version 1.09) [1]. Second step of QC was performed using custom script in Rstudio (version 1.1.383) [2] with R (version 3.4.2) [3] according to plink manufacturers’ instructions. Then, non-autosomal SNPs and variants with deviation from the Hardy-Weinberg equilibrium (HWE) with P<1e-5 were removed. Finally, we checked Genotype Call Rate and removed all variants with completeness <0.99.

## **Sample Quality Control Analysis**

## Similarly, to SNPs QC analysis, we performed two-step QC of samples. Supplementary Information Table S2_2 summarizes the sample quality control (QC) processing steps. First, in GenomeStudio software we removed samples with potential DNA quality and contamination problems. Then, after exporting data to R, we removed samples with inferred sex mismatch and cryptic kinship, according to plink manufacturers’ instructions and KING [4]. Additionally, we checked for heterozygosity rate of autosomal SNPs. The last part of QC was based on EIGENSOFT [5, 6] in order to determine population structure in our samples based on HapMap3 dataset [7]. Samples that failed to qualify as European population (CEU) were removed.

**SUPPLEMENTARY DATA 2 _ TABLES**

**Table S2_1. SNP Quality Control Analysis Results**

| **Step** | **Number Remaining** | **Total Removed** | **Step Number Removed** |
| --- | --- | --- | --- |
| **Total SNPs on Panel** | **298,930** |  |  |
| **1. SNPs with GRCh37 mapping** |  | **699** |  |
| 1.1 Remove chr0 |  |  | 699 |
| **2. Clustering Quality Metrics** | **296,501** | **2,429** |  |
| 2.1 Call Frequency = 0 |  |  | 95 |
| 2.1 AA R Mean ≤ 0.2 |  |  | 268 |
| 2.2 AB R Mean ≤ 0.2 |  |  | 4 |
| 2.3 BB R Mean ≤ 0.2 |  |  | 15 |
| 2.4 Cluster Sep ≤ 0.3 |  |  | 277 |
| 2.5 AA Freq = 1 & AA T Mean > 0.3 |  |  | 66 |
| 2.6 AA Freq = 1 & AA T Dev > 0.06 |  |  | 11 |
| 2.7 BB Freq = 1 & BB T Mean < 0.7 |  |  | 110 |
| 2.8 BB Freq = 1 & BB T Dev > 0.06 |  |  | 30 |
| 2.9 AB T Mean < 0.2 |  |  | 8 |
| 2.10 AB T Mean > 0.8 |  |  | 23 |
| 2.11 10% GC Score ≤ 0.3 |  |  | 823 |
| **3. SNP Errors** | **296,471** | **30** |  |
| 3.2 Duplicate samples ≥2 discordant genotypes |  |  | 30 |
| **4. Hardy-Weinberg equilibrium (HWE) in autosomal SNPs** | **296,370** | **101** |  |
| 4.1 Het Excess > 0.2 |  |  | 48 |
| 4.2 Call Freq < 0.97 & AB_Freq ≥ 0.4 |  |  | 53 |
| **5. Map Filtering** | **286,946** | **9,424** |  |
| 5.1 Remove chrX |  |  | 7947 |
| 5.2 Remove chrM |  |  | 128 |
| 5.3 Remove chrY |  |  | 1349 |
| **6. Allele Frequency** | **286,697** | **249** |  |
| 6.1 HWE P < 1e-5 |  |  | 249 |
| **7. Genotype Call Rates** | **282,876** | **3,821** |  |
| 7.1 Completeness < 0.99 |  |  | 3,821 |
| **Total SNPs Passing QC** | **282,876** |  |  |

**Table S2_2. Sample Quality Control Analysis Results**

| **Step** | **Number Remaining** | **Total Removed** | **Step Number Removed** |
| --- | --- | --- | --- |
| **Probands with -genotype data** | **992** |  |  |
| **Post Genotyping Known Problems** | **934** | **58** |  |
| Genotype problem, call rate <0.5 |  |  | 47 |
| Duplicate samples errors |  |  | 4 |
| Low call rate <0.99 |  |  | 15 |
| **Cryptic QC** | **895** | **39** |  |
| Cryptic duplicates |  |  | 4 |
| Cryptic relationships |  |  | 5 |
| Inferred sex mismatch |  |  | 30 |
| High heterozygosity |  |  | 0 |
| **Ancestry and Substructure QC** | **887** | **8** |  |
| Ancestry outliers |  |  | 8 |
| **Total Proband Samples Passing QC** | **879** | **36** |  |
| Missing phenotype data |  |  | 36 |
| **Total Proband Samples in GWAS** | **843** |  |  |

**References:**

1. Purcell S, Neale B, Todd-Brown K, Thomas L, Ferreira MAR, Bender D, et al. PLINK: A Tool Set for Whole-Genome Association and Population-Based Linkage Analyses. Am J Hum Genet [Internet]. 2007;81(3):559–75. Available from: <http://linkinghub.elsevier.com/retrieve/pii/S0002929707613524>
2. Citing RStudio – RStudio Support [Internet]. [cited 2018 Sep 10]. Available from: <https://support.rstudio.com/hc/en-us/articles/206212048-Citing-RStudio>
3. R: The R Project for Statistical Computing [Internet]. [cited 2018 Sep 10]. Available from: <https://www.r-project.org/>
4. Manichaikul A, Mychaleckyj JC, Rich SS, Daly K, Sale M, Chen WM. Robust relationship inference in genome-wide association studies. Bioinformatics. 2010;26(22):2867–73.
5. Patterson N, Price AL, Reich D. Population structure and eigenanalysis. PLoS Genet. 2006;2(12):2074–93.
6. 27. Price AL, Patterson NJ, Plenge RM, Weinblatt ME, Shadick NA, Reich D. Principal components analysis corrects for stratification in genome-wide association studies. Nat Genet. 2006;38(8):904–9.
7. 28. International T, Consortium H. The International HapMap Project. Nature. 2003;426(6968):789–96.
