# Supplementary material for: Variants influencing age at diagnosis of HNF1A-MODY
Source: Mol Med. 2022 Sep 14;28:113. doi: 10.1186/s10020-022-00542-0 (PMC9476297; doi:10.1186/s10020-022-00542-0)
Supplement: Supplementary file 3 — Additional file 3. Additional data S3. [file 10020_2022_542_MOESM3_ESM.zip › Supp 3/Suplemmentary Data 3.docx]

**Supplementary Data 3 - Top 10 results of GWAS analysis of age at diabetes onset**

| **SNP ID** | **Effective allele** | **Chr** | **Location** | **Nearest gene** | **MAF** | **Est.** | **p-value** |
| --- | --- | --- | --- | --- | --- | --- | --- |
| rs2305198 | C | 10 | Intron | *HK1* | 0.38 | -0.105 | 1.58E-07 |
| rs7079157 | C | 10 | Intron | *HK1* | 0.23 | -0.108 | 2.90E-06 |
| rs10865231 | G | 2 | Intron | *STON1-GTF2A1L* | 0.26 | -0.099 | 3.24E-06 |
| rs349359 | C | 8 | Intron | *KCBN2* | 0.11 | -0.135 | 1.27E-05 |
| rs6899796 | G | 6 | Intergenic | *FOXF2* | 0.36 | -0.08 | 1.62E-05 |
| rs210307 | C | 14 | Intron | *LOC105370504* | 0.23 | 0.095 | 1.83E-05 |
| rs16887775 | G | 4 | Intron | *LOC105374493* | 0.38 | 0.083 | 2.46E-05 |
| rs3828340 | C | 2 | Coding | *STON1-GTF2A1L* | 0.30 | -0.08 | 3.18E-05 |
| rs1166940 | G | 3 | Intron | *ROBO2* | 0.22 | -0.09 | 3.55E-05 |
| rs210318 | C | 14 | Intron | *LOC105370504* | 0.23 | 0.09 | 3.83E-05 |

**Manhattan plot (A) and Q–Q plot (B) from the GWAS analysis of age at diabetes onset.** GWAS, genome-wide association study; Q–Q, quantile–quantile are attached as supplementary data 3 figures.
