# Supplementary figures and images for: Variants influencing age at diagnosis of HNF1A-MODY
Source: Mol Med. 2022 Sep 14;28:113. doi: 10.1186/s10020-022-00542-0 (PMC9476297; doi:10.1186/s10020-022-00542-0)

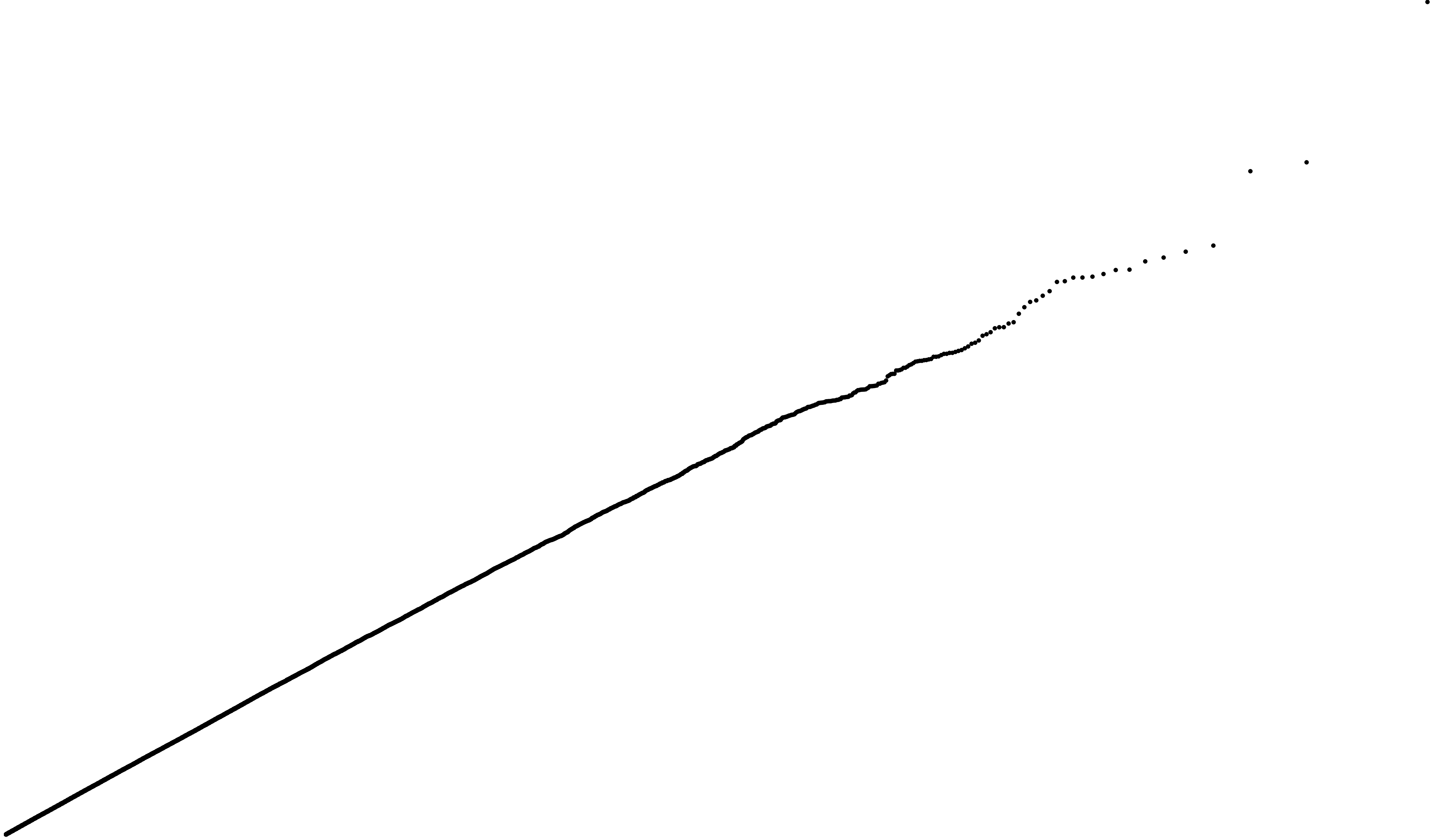

Supplement: Supplementary file 3 — Additional file 3. Additional data S3. [file 10020_2022_542_MOESM3_ESM.zip › Supp 3/Supplementary data figure 3 figure - Q-Q plot.pdf]
