# Supplementary material for: Variants influencing age at diagnosis of HNF1A-MODY
Source: Mol Med. 2022 Sep 14;28:113. doi: 10.1186/s10020-022-00542-0 (PMC9476297; doi:10.1186/s10020-022-00542-0)
Supplement: Supplementary file 4 — Additional file 4. Additional table. [file 10020_2022_542_MOESM4_ESM.docx]

| **AA position** | **Variant** | **ACMG classification** | |
| --- | --- | --- | --- |
| 1 | Met1? | Pathogenic | PVS1, PM2, PP3, PP5 |
| 7 | Gln7X | Pathogenic | PVS1, PM2, PP3, PP5 |
| 9 | Qln9Pro | Uncertain significance | PM2, PP2, PP3 |
| 10 | Thr10Met | Uncertain significance | PP2, PP3, BS2 |
| 15 | Ala15Val | Likely pathogenic | PM2, PM5, PP2, PP3 |
| 16 | Leu16Pro | Uncertain significance | PM2, PP2, PP3 |
| 23 | Lys23fs | Pathogenic | PVS1, PM2, PP3 |
| 24 | Glu24fsdelGA | Pathogenic | PVS1, PM2, PP3 |
| 26 | Leu26Pro | Uncertain significance | PM2, PP2, PP3 |
| 28 | Gln28X | Pathogenic | PVS1, PM2, PP3 |
| 33 | Pro33Leu | Uncertain significance | PM2, PP2, BP4 |
| 44 | Leu44fsdelC | Pathogenic | PVS1, PM2, PP3 |
| 46 | Lys46Thr | Uncertain significance | PM2, PP2, PP3 |
| 54 | Arg54X | Uncertain significance | PM2, PP2, PP3 |
| 54 | Arg54* | Pathogenic | PSV1, PM2, PP3, PP5 |
| 55 | Gly55fs | Pathogenic | PSV1, PM2, PP3 |
| 56 | Glu56* | Pathogenic | PSV1, PM2, PP3 |
| 61 | Val61Gly | Uncertain significance | PM2, PP2, PP3 |
| 62 | Asn62Ser | Uncertain significance | PP2, PP3, BS2 |
| 63 | Gln631del | Pathogenic | PVS1, PM2, PP3 |
| 66 | Glu66fs | Pathogenic | PVS1, PM2, PP3 |
| 75 | Asp75Glu | Uncertain significance | PM2, PP2, PP3 |
| 81 | Phe81fs | Pathogenic | PVS1, PM2, PP3 |
| 95 | Glu95X | Pathogenic | PVS1, PM2, PP3, PP5 |
| 107 | Leu107Arg | Uncertain significance | PM2, PP2, PP3 |
| 108 | Leu108Pro | Uncertain significance | PM2, PP2, PP3 |
| 109 | Gln109X | Pathogenic | PVs1, PM2, PP3 |
| 111 | Asp111Gly | Uncertain significance | PM2, PP2, PP3 |
| 112 | Pro112Leu | Likely pathogenic | PS3, PM2, PP2, PP3 |
| 113 | Trp113Leu | Uncertain significance | PM2, PP2, PP3 |
| 115 | Val115Leu | Uncertain significance | PM2, PP2, PP3 |
| 116 | Ala116Val | Uncertain significance | PM2, PP2, PP3 |
| 116 | Ala116Thr | Uncertain significance | PM2, PP2, PP3 |
| 119 | Val119Ala | Uncertain significance | PM2, PP2, PP3 |
| 123 | Leu123Pro | Uncertain significance | PM2, PP2, PP3 |
| 127 | Asn127del | Uncertain significance | PM2, PP3 |
| 129 | Pro129Thr | Uncertain significance | PM2, PP2, PP3 |
| 130 | Gln130Glu | Uncertain significance | PM2, PP2, PP3 |
| 131 | Arg13Gln | Likely pathogenic | PM2, PM5, PP5, PP2, PP3 |
| 131 | Arg13Trp | Pathogenic | PS3, PM2, PM5, PP2, PP3 |
| 132 | Glu132Lys | Uncertain significance | PM2, PP2, PP3 |
| 133 | Val133Met | Uncertain significance | PM2, PP2, PP3 |
| 135 | Asp135fs | Pathogenic | PVS1, PM2, PP3 |
| 139 | Leu139Pro | Uncertain significance | PM2, PP2, PP3 |
| 140 | Asn140Asp | Uncertain significance | PM2, PP2, PP3 |
| 142 | Ser142Phe | Uncertain significance | PM2, PP2, PP3 |
| 143 | His143Gln | Uncertain significance | PM2, PP2, PP3 |
| 145 | Ser145Phe | Uncertain significance | PM2, PP2, PP3 |
| 146 | Q146L_H147R | Uncertain significance | PM2, PP2, PP3 |
| 146 | Gln146Glu | Uncertain significance | PM2, PP2, PP3 |
| 149 | Arg149Gln | Likely pathogenic | PM2, PM5, PP2, PP3, PP5 |
| 154 | Met154Ile | Uncertain significance | PM2, PP2, PP3 |
| 154 | Met154Val | Uncertain significance | PM2, PP2, PP3 |
| 158 | Lys158_Arg159delinsAsnTrp | Pathogenic | PVS1, PM2, PP3 |
| 158 | Lys158* | Pathogenic | PVS1, PM2, PP3 |
| 158 | Lys158Asn | Uncertain significance | PM2, PP2, PP3 |
| 159 | Arg159Trp | Likely pathogenic | PM2, PM5, PP2, PP3, PP5 |
| 159 | Arg159Gln | Likely pathogenic | PM2, PM5, PP2, PP3, PP5 |
| 163 | Tyr163Asn | Uncertain significance | PM2, PP2, PP3 |
| 166 | Tyr166X | Pathogenic | PVS1, PM2, PP3 |
| 168 | Arg168Pro | Uncertain significance | PM2, PP2, PP3 |
| 170 | Gln170X | Pathogenic | PVS1, PM2, PP3 |
| 171 | Arg171X | Pathogenic | PVS1, PP5, PM2, PP3 |
| 171 | Arg171* | Pathogenic | PVS1, PP5, PM2, PP3 |
| 173 | Val173Met | Uncertain significance | PM2, PP2, PP3 |
| 176 | Gln176* | Pathogenic | PVS1, PM2, PP3, PP5 |
| 176 | Gln176X | Pathogenic | PVS1, PM2, PP3, PP5 |
| 177 | Phe177Ser | Uncertain significance | PM2, PP2, PP3 |
| 184 | Gly184* | Pathogenic | PVS1, PM2, PP3 |
| 187 | Glu187_Asp192del | Pathogenic | PVS1, PM2, PP3 |
| 197 | Thr67Aspfs | Pathogenic | PVS1, PM2, PP3 |
| 200 | Arg200Gln | Pathogenic | PP5, PM2, PM5, PP2, PP3 |
| 200 | Arg200Trp | Likely pathogenic | PM2, PM5, PP2, PP3, PP5 |
| 200 | Arg200Gly | Likely pathogenic | PM2, PM5, PP2, PP3 |
| 203 | Arg203His | Likely pathogenic | PM2, PM5, PP2, PP3 |
| 203 | Arg203Cys | Likely pathogenic | PM2, PM5, PP2, PP3 |
| 203 | Arg203Ser | Likely pathogenic | PM2, PM5, PP2, PP3 |
| 209 | Ala209fs | Pathogenic | PVS1, PM2, PP3 |
| 215 | Phe215fs | Pathogenic | PVS1, PM2, PP3 |
| 218 | Tyr218Cys | Uncertain significance | PM2, PP2, PP3 |
| 220 | Arg220Glyfs | Likely pathogenic | PVS1, PM2 |
| 224 | Pro224Leu | Likely pathogenic | PM2, PM5, PP2, PP3 |
| 225 | Ser225fsdelC | Pathogenic | PVS1, PM2, PP3 |
| 225 | Arg263His | Pathogenic | PVS1, PM2, PP3 |
| 228 | Glu228fs | Pathogenic | PVS1, PM2, PP3 |
| 228 | Glu228Gly | Uncertain significance | PM2, PP2, PP3 |
| 228 | Glu228Lys | Uncertain significance | PM2, PP2, PP3 |
| 229 | Arg229X | Pathogenic | PVS1, PM2, PP2, PP3 |
| 229 | Arg229Pro | Likely pathogenic | PM2, PM5, PP2, PP3 |
| 229 | Arg229* | Pathogenic | PVS1, PM2, PP3, PP5 |
| 229 | Arg229Gln | Likely pathogenic | PM2, PP2, PP3, PP5 |
| 229 | Arg229Gly | Likely pathogenic | PM2, PM5, PP2, PP3 |
| 232 | Leu232Pro | Uncertain significance | PM2, PP2, PP3 |
| 233 | Val233fs | Pathogenic | PVS1, PM2, PP3 |
| 235 | Glu79Glyfs | Pathogenic | PVS1, PM2, PP3 |
| 236 | Cys236* | Pathogenic | PVS1, PM2, PP2, PP3 |
| 238 | Arg238Thr | Pathogenic | PVS1, PM2, PM5, PP2, PP3 |
| 240 | Glu240Gln | Uncertain significance | PM2, PP2, PP3 |
| 241 | Cys241Tyr | Uncertain significance | PM2, PP2, PP3 |
| 244 | Arg244* | Pathogenic | PVS1, PM2, PP3 |
| 246 | Val246Leu | Uncertain significance | PM2, PP2, PP3 |
| 247 | Ser247fs | Pathogenic | PVS1, PM2, PP3 |
| 247 | c.732_733del | Pathogenic | PVS1, PM2, PP3 |
| 249 | Ser249Pro | Uncertain significance | PM2, PP2, PP3 |
| 253 | Gly253Arg | Uncertain significance | PM2, PP2,PP3 |
| 253 | G253G | Uncertain significance | PM2, PP3, BP7 |
| 257 | Asn257Thr | Uncertain significance | PM2, PP2, PP3 |
| 259 | Val259Phe | Uncertain significance | PM2, PP2, PP3 |
| 260 | Thr260Met | Pathogenic | PP5, PM1, PM2, PP2, PP3 |
| 260 | Thr260Ala | Likely pathogenic | PM1, PM2, PM5, PP2, PP3 |
| 261 | Glu261Lys | Likely pathogenic | PM1, PM2, PP2, PP3 |
| 263 | Arg263Cys | Likely pathogenic | PM1, PM2, PM5, PP2,PP3, PP5 |
| 263 | Arg263His | Pathogenic | PP5, PM1, PM2, PM5, PP2, PP3 |
| 266 | Asn266Lys | Likely pathogenic | PM1, PM2, PP2, PP3 |
| 267 | Trp267Arg | Likely pathogenic | PM1, PM2, PP2, PP3 |
| 267 | Trp267X | Pathogenic | PVS1, PM2, PP3 |
| 267 | Trp267* | Pathogenic | PVS1, PM2, PP3 |
| 270 | Asn270Ser | Likely pathogenic | PM1, PM2, PP2, PP3 |
| 271 | Arg271Trp | Likely pathogenic | PM1, PM2, PM5, PP5, PP2, PP3 |
| 271 | Arg271Gly | Likely pathogenic | PM1, PM2, PM5, PP2, PP3 |
| 271 | Arg271fs | Pathogenic | PVS1, PM2, PP3 |
| 272 | Arg272His | Pathogenic | PP5, PM1, PM2, PM5, PP2, PP3 |
| 272 | Arg272Cys | Likely pathogenic | PM1, PM2, PM5, PP2, PP3, PP5 |
| 273 | Lys273Asn | Likely pathogenic | PM1, PM2, PP2, PP3 |
| 275 | Glu275Val | Likely pathogenic | PM1, PM2, PP2, PP3, PP5 |
| 275 | Glu275del | Likely pathogenic | PM1, PM2, PP2, PP3 |
| 276 | Ala276Asp | Pathogenic | PS3, PM1, PM2, PM5, PP2, PP3 |
| 278 | Arg278Gln | Uncertain significance | PM1, PP2, PP3, BS2 |
| 280 | Lys280Glu | Likely pathogenic | PM1, PM2, PP2, PP3 |
| 286 | Tyr286Tfs*57 | Likely pathogenic | PVS1, PM2 |
| 288 | Gly288Trp | Benign | PP2, PP3, BS1, BS2 |
| 289 | Phe289Arg | Uncertain significance | PM2, PP2, PP3 |
| 289 | Phe289fs | Pathogenic | PVS1, PM2, PP3 |
| 289 | Pro289Leu | Uncertain significance | PM2, PP2, PP3 |
| 291 | Pro291fs | Pathogenic | PVS1, PM2, PP3, PP5 |
| 292 | Gly292fs | Pathogenic | PVS1, PP5, PP3 |
| 292 | Gly292Argfs*26 | Pathogenic | PVS1, PP5, PP3 |
| 322 | Tyr322Cys | Benign | PP2, PP3, BS1, BS2 |
| 328 | Ser328Arg | Uncertain significance | PM2, PP2, PP3 |
| 335 | Ser335X | Pathogenic | PVS1, PM2, PP3 |
| 341 | Leu341X | Pathogenic | PVS1, PM2, PP3 |
| 343 | Thr343fs | Pathogenic | PVS1, PM2, PP3 |
| 350 | Gln350fs | Pathogenic | PVS1, PM2, PP3 |
| 351 | Val351fs | Pathogenic | PVS1, PM2, PP3 |
| 379 | Pro379Ala | Benign | PP2, PP3, BS1, BS2 |
| 379 | Pro379Thr | Uncertain significance | PM5, PP2, PP3, BS2 |
| 379 | Pro379Argfs*40 | Pathogenic | PVS1, PM2, PP3, PP5 |
| 380 | Val380fs | Pathogenic | PVS1, PP5, PM2 |
| 383 | Leu383Alafs*4 | Pathogenic | PVS1, PM2, PP3 |
| 384 | Thr384Ifs | Pathogenic | PVS1, PP5, PM2 |
| 384 | Thr384Lys | Uncertain significance | PM2, PP2, PP3 |
| 399 | Gln399* | Pathogenic | PVS1, PM2, PP3 |
| 408 | Leu408Ile | Uncertain significance | PM2, PP2, PP3 |
| 412 | Met412Thr | Uncertain significance | PM2, PP2, PP3 |
| 414 | Ile414fs | Pathogenic | PVS1, PM2, PP3 |
| 418 | Glu418X | Pathogenic | PVS1, PM2, PP3 |
| 437 | Gly437Val | Likely pathogenic | PP3, PM2, PP2 |
| 442 | Gln442fs | Pathogenic | PVS1, PM2, PP3 |
| 443 | Ala443fs | Pathogenic | PVS1, PM2, PP3 |
| 444 | Gln444fs | Pathogenic | PVS1, PM2, PP3, PP5 |
| 445 | Ser445fs | Pathogenic | PVS1, PM2, PP3 |
| 447 | Pro447Leu | Likely pathogenic | PP5, PM2, PP2, PP3 |
| 453 | Gly453fs | Pathogenic | PVS1, PM2, PP3 |
| 457 | Thr457Ile | Uncertain significance | PM2, PP2, PP3 |
| 460 | Cys460fs | Uncertain significance | PM2, PM4, BP4 |
| 463 | Gln463fs | Pathogenic | PVS1, PM2, PP3 |
| 463 | Gln463X | Pathogenic | PVS1, PM2, PP3 |
| 465 | Ser465Tyr | Uncertain significance | PM2, PP2, PP3 |
| 466 | Gln466X | Pathogenic | PVS1, PM2, PP3, PP5 |
| 467 | Pro467Leu | Uncertain significance | PM2, PP2, PP3 |
| 474 | Gln474* | Pathogenic | PVS1, PM2, PP3 |
| 475 | Pro475fs | Pathogenic | PVS1, PM2, PP3 |
| 483 | His483Arg | Uncertain significance | PM2, PP2, PP3 |
| 488 | Pro488Thr | Uncertain significance | PM2, PP2, PP3 |
| 492 | Thr492Ile | Uncertain significance | PM2, PP2, PP3 |
| 495 | Gln495Leu | Uncertain significance | PM2, PP2, PP3 |
| 501 | Ala501Thr | Likely pathogenic | PVS1, PM2, PP2, PP3 |
| 508 | Glu508Val | Uncertain significance | PM2, PP2, PP3 |
| 513 | Thr513Ser | Uncertain significance | PM2, PP2, BP4 |
| 514 | His514Arg | Benign | PP2, PP3, BS1, BS2 |
| 516 | Asp526Tyr | Uncertain significance | PM2, PP2, PP3 |
| 519 | Pro519Leu | Uncertain significance | PM2, PP2, PP3 |
| 526 | Asp526Asn | Uncertain significance | PP2, PP3, BS2 |
| 528 | Tyr528Iso | Uncertain significance | PM2, PP2, BP4 |
| 529 | Asn529fs | Pathogenic | PVS1, PM2, PP3 |
| 532 | Ala532Thr | Uncertain significance | PM2, PP2, BP4 |
| 546 | Asp546fs | Pathogenic | PSV1, PM2, PP3 |
| 546 | Asp546Gly | Uncertain significance | PM2, PP2, PP3 |
| 551 | Leu551Ser | Benign | BA1, BP4 |
| 573 | Ala573fs | Likely pathogenic | PVS1, PM2 |
| 583 | Arg583Gln | Benign | PP2, PP3, BS1, BS2, BS3 |
| 587 | Ser587fs | Pathogenic | PVS1, PM2, PP3 |
| 594 | Ser594Ile | Uncertain significance | PM2, PP2, PP3 |
| 602 | Asp602fs | Pathogenic | PVS1, PM2, PP3 |
| 608 | Ser608fs | Pathogenic | PVS1, PM2, PP3 |
| 618 | Ile618fs | Pathogenic | PVS1, PM2, PP3 |
| 620 | Thr620Ile | Likely pathogenic | PM2, PP2, PP3, PP5 |
| non coding | IVS8nt+1G>A | Pathogenic | PVS1, PM2, PP3 |
| non coding | IVS7-6G>A | Uncertain significance | PM2, PP5, BP4 |
| non coding | c.526+1G>A | Pathogenic | PVS1, PM2, PP3, PP5 |
| non coding | c.526+1G>C | Pathogenic | PVS1, PM2, PP3 |
| non coding | N/IVS7-6G>A | Uncertain significance | PM2, PP5, BM4 |
| non coding | c.1107+1G>A | Pathogenic | PVS1, PM2, PP3 |
| non coding | c.1502-6G>A | Uncertain significance | PM2, PP5, BP4 |
| non coding | IVS1+1G>T | Pathogenic | PVS1, PM2, PP3 |
| non coding | IVS4nt-2A>G | Pathogenic | PVS1, PM2, PP3 |
| non coding | c.1502-2A>T | Pathogenic | PVS1, PM2, PP3 |
| non coding | c.526+2delT | Pathogenic | PVS1, PM2, PP3 |
| non coding | IVS2nt+1G>A | Pathogenic | PVS1, PM2, PP3, PP5 |
| non coding | c.1-?_1893+?del | Pathogenic |  |
| non coding | c.1501G>A | Likely pathogenic | PVS1, PM2, PP2, PP3 |
| non coding | c.1623+1G>A | Pathogenic | PVS1, PM2, PP3 |
| non coding | c.1768+1G>C | Pathogenic | PVS1, PM2, PP3 |
| non coding | c.327-?_1893+?del | Pathogenic |  |
| non coding | c.713+1G>A | Likely pathogenic | PVS1, PM2, PP2, PP3 |
| non coding | c.714-1G>A | Pathogenic | PVS1, PS3, PM2, PP3 |
| non coding | c.955+2T>C | Pathogenic | PVS1, PM2, PP3 |
| non coding | IVS5-1del TAG | Pathogenic | PVS1, PM2, PP3 |
| non coding | c.-176G>C | Pathogenic | PVS1, PM2, PP3, PP5 |
| non coding | c.1-?_326+?del | Pathogenic |  |
| non coding | c.327-1G>A | Pathogenic | PVS1, PM2, PP3 |
| non coding | c.526+5G>A | Uncertain significance | PM2 |
| non coding | c.527-?_1309+?del | Pathogenic |  |
| non coding | c.955+4_955+5insA | Uncertain significance | PM2, PP3 |
| non coding | c.955+5G>C | Uncertain significance | PM2 |
| non coding | c.956-2A>G | Pathogenic | PVS1, PM2, PP3 |
